# Frequency of Thiopurine S-methyltransferase (TPMT) Genotypes Among Patients With Acute Lymphoblastic Leukemia at a Tertiary Care Hospital in Bangladesh: A Cross-Sectional Study

**DOI:** 10.7759/cureus.117511

**Published:** 2026-10-05

**Authors:** Md Zakaria Hossen, Md Foys Ullah, Nahid Sultana, Md Gulam Kibria, Akhil Ranjan Biswas

**Affiliations:** 1 Department of Haematology, Rajshahi Medical College Hospital, Rajshahi, BGD; 2 Department of Haematology and Bone Marrow Transplantation, Continental Hospital, Dhaka, BGD; 3 Department of Haematology, Dhaka Medical College Hospital, Dhaka, BGD; 4 Department of Haematology, Faridpur Medical College Hospital, Faridpur, BGD; 5 Department of Haematology, Chittagong Medical College Hospital, Chattogram, BGD

**Keywords:** 6-mercaptopurine, acute lymphoblastic leukemia, bangladesh, intermediate metabolism, pharmacogenetics, thiopurine s-methyltransferase, tpmt

## Abstract

Background: 6-Mercaptopurine is central to consolidation and maintenance therapy in acute lymphoblastic leukemia (ALL). Thiopurine S-methyltransferase (TPMT) inactivates its cytotoxic thioguanine nucleotides, and loss-of-function TPMT variants predispose carriers to severe myelosuppression at conventional doses. Published Bangladeshi evidence is confined to children. This study determined the frequency and distribution of laboratory-reported TPMT diplotypes and the corresponding predicted metabolizer phenotypes among Bangladeshi patients with ALL.

Methods: This cross-sectional study was conducted in the Department of Hematology and Bone Marrow Transplantation Unit, Dhaka Medical College Hospital, Dhaka, from February 2023 to July 2024. All eligible, consenting patients with newly diagnosed ALL were enrolled consecutively, and 40 were analyzed. Genotyping was outsourced to two commercial laboratories, which sequenced complementary DNA reverse transcribed from peripheral blood RNA rather than the genomic DNA specified by international recommendations; the assay was therefore nonstandard and unvalidated, and haplotype phasing was not performed. Reported diplotypes were translated into predicted metabolizer phenotypes. Exact binomial 95% confidence intervals (CIs) were calculated, and association analyses were post hoc and exploratory, using two-sided exact tests.

Results: Among the participants, 31 (77.5%) were male patients, and 24 (60.0%) had B-lineage ALL. Thirty-eight carried TPMT*1/*1 and were predicted normal metabolizers (95.0%; exact 95% CI, 83.1-99.4), and two were predicted intermediate metabolizers (5.0%; 0.6-16.9), reported as TPMT*1/*3A and TPMT*1/*3C. No poor metabolizer was identified on the diplotypes as reported (0/40; 0.0-8.8). Because the phase was not established, the patient reported as TPMT*1/*3A could instead carry TPMT*3B and TPMT*3C in trans and would then be a poor metabolizer requiring an approximately 10-fold rather than twofold dose reduction, leaving one intermediate and one poor metabolizer. Exploratory tests detected no difference by sex, immunophenotypic subtype, or hematological parameters, but had no meaningful power.

Conclusions: In this study, two of 40 Bangladeshi patients with ALL carried a reported reduced-function TPMT diplotype. Because every call derived from a nonstandard, unvalidated RNA-based assay and the TPMT*3A phase remains unresolved, these estimates are provisional and require DNA-based, phased confirmation. They nonetheless support international guidance favoring pretreatment TPMT genotyping. Combined NUDT15 testing is indicated by published Asian evidence rather than by these data, since NUDT15 was not assessed. Larger multicenter studies with prospective toxicity follow-up are needed.

## Introduction

Acute lymphoblastic leukemia (ALL) is an aggressive hematological malignancy that originates from the clonal proliferation of maturation-arrested lymphoid progenitors in the bone marrow, with dissemination to peripheral blood, lymphoid organs, and sanctuary sites, including the central nervous system and testes [1]. The disease peaks in early childhood and again in later adult life, and although contemporary pediatric protocols cure most children, survival among adults remains substantially lower [1,2]. The global burden is expanding rather than contracting. Global burden of disease estimates show that the number of incident ALL cases increased between 1990 and 2021, even as age-standardized mortality declined [3], and the residual burden of leukemia is shifting toward low- and middle-income countries [4]. In Bangladesh, ALL accounts for 14.1% of more than 5,000 hematological malignancies diagnosed across 10 specialized centers and for more than one-third of such malignancies in patients younger than 20 years of age [5,6]; elsewhere, male patients consistently outnumber female patients [7].

The curative effect in ALL patients depends less on achieving remission than on delivering prolonged antimetabolite therapy without interruption. 6-Mercaptopurine (6-MP) is the backbone of consolidation and interim maintenance, and it is taken daily for two to three years, with outcomes closely tied to sustained dose intensity [8,9]. It is an inactive prodrug: intracellular phosphorylation generates cytotoxic thioguanine nucleotides that are ultimately incorporated into DNA, whereas thiopurine S-methyltransferase (TPMT) diverts the drug into inactive methylated metabolites [8,10]. Loss-of-function TPMT variants, principally TPMT*2, *3A, *3B, and *3C, reduce or abolish this methylation so that carriers are classified as normal, intermediate, or poor metabolizers [11]. Enzyme activity is inherited as a codominant trait, and the variant panels required for clinical testing are now standardized internationally [12]. Guidelines converge on reducing the starting dose of 6-MP by roughly half in intermediate metabolizers and approximately 10-fold in poor metabolizers and now regard genotyping before the first thiopurine dose as essential rather than optional [11-13].

The clinical stakes of not knowing a patient’s genotype are highest precisely where our patients are treated. During consolidation, 6-MP is given alongside several other myelosuppressive agents, so a decreased blood count cannot be attributed to any single drug, and serial complete blood counts cannot be used as a substitute for pharmacogenetic information. Reduced-function genotypes measurably lower the dose that patients can tolerate [14], and meta-analyses report several-fold increases in leukopenia risk among carriers [15]. Variant frequencies differ markedly between populations, so estimates generated in European or East Asian cohorts cannot be safely assumed for South Asian patients [16-18]. Bangladeshi data remain sparse. A study of 75 children with ALL reported a TPMT*3C allele frequency of 1.0% [19], whereas other local thiopurine pharmacogenetic evidence comes from adults with systemic lupus erythematosus [20,21] and population screening for NUDT15 [22]. TPMT testing has not entered routine oncology practice in Bangladesh [23]. Locally generated data are therefore needed to inform feasible pretreatment testing strategies.

The primary objective of the present study was therefore to determine the frequency and distribution of laboratory-reported TPMT diplotypes, and of the corresponding predicted metabolizer phenotypes, among patients with ALL attending a tertiary care hospital in Bangladesh. Distribution here means the way the observed diplotypes were partitioned across the normal, intermediate and poor metabolizer categories defined below, and no analytical claim beyond that partition is intended. A secondary, post hoc exploratory analysis examined whether the predicted metabolizer status appeared to differ by sex, immunophenotypic subtype, or presenting hematological parameters. No prior mechanistic or epidemiological hypothesis links germline TPMT variation to these variables, and no such association was prespecified; that analysis was undertaken only to describe how the identified carriers were situated within the cohort and is reported for completeness rather than as a test of any hypothesis.

## Materials and methods

Study population

This cross-sectional observational study was conducted in the Department of Hematology and Bone Marrow Transplantation Unit of Dhaka Medical College Hospital, Dhaka, Bangladesh, over 18 months, from February 2023 to July 2024. Patients admitted to the department or attending its outpatient service with newly diagnosed ALL were considered for enrollment. The diagnosis of ALL was established by peripheral blood film examination, bone marrow morphology, and immunophenotyping. A new diagnosis of ALL established on those grounds was the sole inclusion criterion, and no age limit was applied. We excluded patients who declined participation, were receiving palliative treatment only, or did not plan to receive a multidrug consolidation regimen containing 6-MP with other myelosuppressive agents. Sampling was purposive in that recruitment was confined to this single department and the study period rather than drawn from a defined population frame, but within that frame it was consecutive and complete. Every patient given a new diagnosis of ALL in the department during the period was assessed against the criteria above as part of the routine diagnostic work-up, and every patient who met them was invited in the order of presentation until the period closed. Selection was therefore determined only by presentation, eligibility, and consent, and no eligible patient was passed over on any clinical, laboratory, or genotype-related grounds. Because molecular facilities were unavailable at the study site, genotyping was outsourced, and its cost was borne by the participants, so willingness and ability to meet that cost also determined enrollment in practice. Forty patients were enrolled on this basis, and all 40 yielded an interpretable laboratory report and were analyzed, with no withdrawals and no exclusions after enrollment. A separate log of patients assessed but not enrolled was not maintained, so the number who were ineligible or who declined cannot be reported, and this is acknowledged as a limitation.

Data collection procedure

Data were collected via a semistructured questionnaire and a structured investigation checklist. Information on sex, immunophenotypic subtype, and baseline hematological parameters was recorded. The hematological parameters included total leucocyte count, hemoglobin concentration and platelet count. The collected information was checked for completeness, accuracy, and internal consistency before analysis. Ethical approval was obtained from the Ethical Review Committee of Dhaka Medical College, and permission was obtained from the concerned department before enrollment. The nature, purpose, possible risks, and potential benefits of the study were explained to each participant, and written informed consent was obtained before data and blood sample collection. The confidentiality of all the study records was maintained throughout the study.

Sample size determination

The sample size was initially planned for the estimation of a single population proportion with the formula $n = \frac{Z^2pq}{e^2}$, taking the anticipated frequency of heterozygous TPMT variants as 2.76% from a southern Indian population [24], so that p was 0.0276, q was 0.9724, Z was 1.96 for a 95% confidence level, and the allowable error was 0.05; the calculated value was 41.25, which was rounded to 42. On review during revision, we recognize that this calculation is not appropriate for the present research question because an absolute allowable error of 0.05 is larger than the anticipated proportion itself, so the implied interval extends below zero, and the nominal target of 42 does not correspond to any meaningful level of precision. For a rare variant, the absolute margin must be smaller than the expected proportion, and estimating a proportion of approximately 3% with an absolute margin of 0.02 would require more than 250 participants, which was not achievable within the resources and caseload of a single department over the study period. The achieved sample of 40 is accordingly best described as a feasibility-limited consecutive series comprising all eligible and consenting patients who presented during the defined period, and no formal precision target was met. Precision is therefore reported directly through the exact binomial CIs that accompany each estimate, which for the intermediate-metabolizer frequency of 5.0% extend from 0.6% to 16.9%, and this imprecision is carried forward into the interpretation of every result.

TPMT genotyping

Molecular genotyping facilities were not available at the study site. Approximately 4 mL of peripheral blood was collected from each participant in an ethylenediaminetetraacetic acid tube under the supervision of the study guide and was transported promptly, with the precautions specified by the receiving laboratories, to two commercial molecular laboratories in Dhaka, DNA Solution Ltd. (Dhaka, Bangladesh) and The Lal Path (Dhaka, Bangladesh), which performed all genotyping and issued the diplotype reports. RNA was manually extracted from peripheral blood according to the manufacturer’s instructions and stored at -80°C until further processing. Complementary DNA was prepared with a ProtoScript cDNA synthesis kit (New England Biolabs, Ipswich, MA) according to the manufacturer’s instructions. The target region was subsequently amplified via gene-specific primers and AccuPower PCR PreMix (Bioneer, Korea) on an ABI 2720 thermal cycler (Applied Biosystems, Foster City, CA). Cycle sequencing was performed via a BigDye Terminator v3.0 kit (Applied Biosystems), followed by capillary sequencing via a Genetic Analyzer 3500Dx (Applied Biosystems). The generated sequences were reviewed, and the laboratory results were reported within five to seven days. The laboratories stated that genotyping followed internationally accepted procedures and that diplotypes were assigned according to universal star allele reporting criteria, and each returned a diplotype call rather than primary sequence data. Beyond the platform details given above, the contracted laboratories did not release their assay documentation, and we are therefore unable to report the RNA extraction kit, the primer sequences, the amplicon length, the exons or transcript region covered, the specific set of TPMT variants the assay was designed to interrogate, the run-level positive and negative control results, the RNA concentration, purity and integrity values, or the software, reference sequence, and electropherogram quality criteria used for variant calling. What can be stated with certainty is that no variant outside the amplified region and no variant absent from mature messenger RNA could have been detected, that no sample was genotyped in duplicate or on an independent aliquot, and that no call was confirmed by a second, DNA-based method. These gaps are limitations of the study and are set out again in the Strengths and Limitations section. The laboratory-reported TPMT diplotypes were translated into predicted metabolizer phenotypes according to established consensus definitions [12]. Two features of this approach require explicit acknowledgment. First, current recommendations from the Clinical Pharmacogenetics Implementation Consortium, the Dutch Pharmacogenetics Working Group, and the Association for Molecular Pathology specify genomic DNA as the template for clinical TPMT genotyping [11-13]. Complementary DNA derived from peripheral blood RNA was used here because it was the platform the contracted laboratories offered at the time of the study and because sequencing the transcript covers the coding positions that define the common reduced-function star alleles. We are aware of no published validation of RNA-based TPMT genotyping against DNA-based genotyping; none was undertaken here, and allele-specific differences in transcript abundance or degradation could, in principle, distort detection of a heterozygous variant. Every genotype call in this study should therefore be read as the output of a nonstandard and unvalidated assay rather than as a clinically actionable genotype. Second, haplotype phasing was performed neither by the laboratories nor within this study. TPMT*3A is defined by c.460G>A and c.719A>G in cis on one allele, whereas the same two variants in trans correspond to TPMT*3B and TPMT*3C on opposite alleles and to a poor-metabolizer phenotype [12]. The laboratory-reported TPMT*1/*3A assignment is consequently unresolved between intermediate and poor metabolism, a distinction that carries an approximately 10-fold difference in the recommended starting dose, and it is treated as unresolved throughout this report.

Definition of the predicted TPMT metabolizer status

Participants carrying two normal-function TPMT alleles, represented by TPMT*1/*1, were classified as normal metabolizers. Participants carrying one normal-function allele and one no-function allele, such as TPMT*1/*2, TPMT*1/*3A, TPMT*1/*3B, TPMT*1/*3C, or TPMT*1/*4, were classified as intermediate metabolizers. A possible intermediate metabolizer was defined as an individual carrying one uncertain-function allele together with one no-function allele. Participants carrying two nonfunction alleles were classified as poor metabolizers. Diplotype combinations involving two uncertain-function alleles or one normal-function allele and one uncertain-function allele were classified as indeterminate [12].

Statistical analysis

The data were analyzed via IBM SPSS Statistics version 25.0 (IBM Corp., Armonk, NY). Categorical variables are presented as frequencies and percentages. Exact binomial 95% CIs were calculated for the estimated frequencies of the predicted normal, intermediate, and poor TPMT metabolizers. Two-sided Fisher’s exact test was used to explore differences in predicted TPMT metabolism status according to sex and immunophenotypic subtype. The Fisher-Freeman-Halton exact test was used to evaluate differences across categories of total leucocyte count, hemoglobin concentration, and platelet count. All association analyses were considered exploratory because only two intermediate metabolizers were identified. A two-sided p value less than 0.05 was considered statistically significant. For the comparisons, the Pearson chi-square statistic and its degrees of freedom were additionally computed from the same contingency tables and are reported alongside the corresponding exact p values; because several expected cell counts were below five, statistical inference was based on the exact tests rather than on the asymptotic chi-square distribution. The association analyses were post hoc and exploratory. They were not prespecified, no adjustment for multiple comparisons was applied, and with only two carriers, the tests have essentially no power to detect a difference of any magnitude. The resulting p values are descriptive only, and no inference about the presence or the absence of an association should be drawn from them.

Ethics

The study was approved by the Ethical Review Committee of Dhaka Medical College, Dhaka, Bangladesh (approval no: ERC-DMC/ECC/2023/17). Written informed consent was obtained from every participant before enrollment. All procedures were performed in accordance with the Declaration of Helsinki.

## Results

Among the participants, 31 (77.5%) were male and nine (22.5%) were female patients. B-lineage ALL was present in 24 patients (60%), and T-lineage ALL was present in 16 patients (40%). At presentation, 15 patients (37.5%) had a total leucocyte count above 11,000-50,000/mm³, and 13 (32.5%) had a total leucocyte count above 50,000-100,000/mm³. The hemoglobin concentration exceeded 9 g/dL in 18 patients (45%), and 17 patients (42.5%) had a platelet count below 25,000/mm³. The baseline profile of the cohort is presented in Table 1.

**Table 1:** Baseline characteristics of the study participants (n = 40) Data are presented as frequencies and percentages. This table is descriptive; no statistical test was applied, and therefore no test statistic or p value is reported ALL: acute lymphoblastic leukemia

Variable	Value, n (%)
Sex	
Male	31 (77.5)
Female	9 (22.5)
Immunophenotypic subtype	
B-lineage ALL	24 (60.0)
T-lineage ALL	16 (40.0)
Total leucocyte count (/mm³)	
4,000-11,000	4 (10.0)
>11,000-50,000	15 (37.5)
>50,000-100,000	13 (32.5)
>100,000	8 (20.0)
Hemoglobin (g/dL)	
<7	9 (22.5)
7-9	13 (32.5)
>9	18 (45.0)
Platelet count (/mm³)	
<25,000	17 (42.5)
25,000-50,000	6 (15.0)
>50,000-75,000	3 (7.5)
>75,000-100,000	3 (7.5)
>100,000	11 (27.5)

Thirty-eight patients carried TPMT*1/*1 and were classified as having normal metabolism (95.0%; exact 95% CI, 83.1-99.4). Two patients were classified as having predicted intermediate metabolism (5.0%; exact 95% CI, 0.6-16.9). The laboratory reported one as TPMT*1/*3A and the other as TPMT*1/*3C. On the diplotypes as reported, no poor metabolizer was identified (0/40; exact 95% CI, 0.0-8.8). Because the phase of c.460G>A and c.719A>G was not established in the patient reported as TPMT*1/*3A, an alternative configuration of TPMT*3B and TPMT*3C in trans cannot be excluded; under that configuration the cohort would comprise 38 normal metabolizers, one intermediate metabolizer (2.5%; exact 95% CI, 0.1-13.2) and one poor metabolizer (2.5%; exact 95% CI, 0.1-13.2). Both configurations are carried forward in the interpretation. The distribution is presented in Table 2.

**Table 2:** Distribution of laboratory-reported TPMT diplotypes and predicted metabolizer status (n = 40) The aggregate intermediate-metabolizer frequency was 2/40 (5.0%; exact 95% CI, 0.6-16.9). No poor metabolism was observed (0/40; exact 95% CI, 0.0-8.8). Diplotypes are those reported by the external laboratory; haplotype phasing was not documented. Exact 95% confidence intervals were calculated by the Clopper-Pearson exact binomial method TPMT: thiopurine S-methyltransferase; CI: confidence interval

Reported TPMT diplotype	Predicted phenotype	n (%)	Exact 95% CI
TPMT*1/*1	Normal metabolizer	38 (95.0)	83.1-99.4
TPMT*1/*3A	Intermediate metabolizer (phase not established; poor metabolizer not excluded)	1 (2.5)	0.1-13.2
TPMT*1/*3C	Intermediate metabolizer	1 (2.5)	0.1-13.2

The analyses reported in this and the following subsection were post hoc and exploratory, were not prespecified, and are presented for descriptive completeness only; with two carriers, they cannot support any inference. Both intermediate metabolizers were male; no statistically significant difference in predicted metabolizer status was detected by sex (exact p = 1.000). One patient with B-lineage ALL (4.2%) and one with T-lineage ALL (6.3%) had intermediate metabolism, and no difference was detected according to the immunophenotypic subtype (exact p = 1.000). The distributions are summarized in Table 3. The corresponding Pearson chi-square values were 0.611 (df = 1) for sex and 0.088 (df = 1) for immunophenotypic subtype.

**Table 3:** Association of sex and immunophenotypic subtype with predicted TPMT metabolizer status (n = 40) Exact p values were derived from two-sided Fisher’s exact tests. Percentages are calculated within each row category. These comparisons were post hoc and exploratory, were not prespecified, and are presented for descriptive completeness. With only two carriers, they have no meaningful power, and no inference should be drawn from the p values ALL: acute lymphoblastic leukemia; TPMT: thiopurine S-methyltransferase

Variable	Normal metabolizer, n (%)	Intermediate metabolizer, n (%)	Test statistic	Exact p value
Sex				
Male	29 (93.5)	2 (6.5)	χ² = 0.611 (df = 1)	1.000
Female	9 (100)	0 (0)		
Subtype				
B-lineage ALL	23 (95.8)	1 (4.2)	χ² = 0.088 (df = 1)	1.000
T-lineage ALL	15 (93.8)	1 (6.3)		

Both intermediate metabolizers had a total leucocyte count above 50,000-100,000/mm³, representing 15.4% of that category; no statistically significant difference in predicted metabolizer status was detected across leucocyte count categories (exact p = 0.328). One intermediate metabolizer had a hemoglobin concentration below 7 g/dL, and the other had a hemoglobin concentration above 9 g/dL (exact p = 0.700). One patient had a platelet count of 25,000-50,000/mm³, and the other had a count above 100,000/mm³ (exact p = 0.455). These exploratory results are shown in Table 4. The corresponding Pearson chi-square values were 4.372 (df = 3) for total leucocyte count, 1.404 (df = 2) for hemoglobin concentration, and 3.317 (df = 4) for platelet count.

**Table 4:** Association of baseline hematological parameters with predicted TPMT metabolizer status (n = 40) Exact p values were derived from the Fisher-Freeman-Halton test. Percentages are calculated within each row category. These comparisons were post hoc and exploratory, were not prespecified, and are presented for descriptive completeness. With only two carriers, they have no meaningful power, and no inference should be drawn from the p values TPMT: thiopurine S-methyltransferase

Parameter	Normal metabolizer, n (%)	Intermediate metabolizer, n (%)	Test statistic	Exact p value
Total leucocyte count (/mm³)				
4,000-11,000	4 (100)	0 (0)	χ² = 4.372 (df = 3)	0.328
>11,000-50,000	15 (100)	0 (0)		
>50,000-100,000	11 (84.6)	2 (15.4)		
>100,000	8 (100)	0 (0)		
Hemoglobin (g/dL)				
<7	8 (88.9)	1 (11.1)	χ² = 1.404 (df = 2)	0.700
7-9	13 (100)	0 (0)		
>9	17 (94.4)	1 (5.6)		
Platelet count (/mm³)				
<25,000	17 (100)	0 (0)	χ² = 3.317 (df = 4)	0.455
25,000-50,000	5 (83.3)	1 (16.7)		
>50,000-75,000	3 (100)	0 (0)		
>75,000-100,000	3 (100)	0 (0)		
>100,000	10 (90.9)	1 (9.1)		

## Discussion

In this single-center cohort of 40 Bangladeshi patients with ALL, 38 were predicted to be normal TPMT metabolizers (95.0%; exact 95% CI, 83.1-99.4), and two were predicted to be intermediate metabolizers (5.0%; exact 95% CI, 0.6-16.9). The laboratory reported one of these as TPMT*1/*3A and the other as TPMT*1/*3C. No poor metabolizer was identified on the diplotypes as reported, although the exact 95% CI for a zero count extends to 8.8%, and the unresolved phase of the TPMT*3A call leaves open the possibility that one patient in this cohort is in fact a poor metabolizer. The descriptive implication is that a small proportion of patients treated in this setting may carry a reduced-function TPMT allele and may require a lower starting dose of 6-MP with closer titration. Because every genotype call rests on a nonstandard, RNA-derived, and unvalidated assay, this implication is provisional and is offered as a reason to establish DNA-based pretreatment testing rather than as a validated frequency estimate.

A previous pediatric study of 75 Bangladeshi children with ALL reported a TPMT*3C allele frequency of 1.0% [19]. Our cohort also included one TPMT*1/*3C carrier, whereas the laboratory reported another patient with TPMT*1/*3A. Direct comparisons are limited by differences in age groups, assay approaches, and frequency metrics. In neighboring populations, TPMT*3C and TPMT*3B were detected in a Sri Lankan cohort at frequencies of 1.9% and 0.2%, respectively [17], whereas North Indian data identified TPMT variants in 4.40% of individuals, with TPMT*3A, TPMT*3B, and TPMT*3C represented [18]. In a large multidisease cohort, the wild-type genotype was present in 90.8%, the heterozygote genotype was present in 9.0%, and the homozygous variant carrier genotype was present in 0.22% of patients [25]. Our imprecise estimate of 5.0% lies within the broad range reported across these populations, but it should not be interpreted as a national prevalence estimate.

The reported allelic pattern deserves comment. TPMT*3C is the variant most often described in Asian and African populations, whereas TPMT*3A predominates in individuals of European ancestry [12,16]. Taken at face value, the presence of both reported diplotypes would suggest that a panel restricted to TPMT*3C could miss carriers. That inference cannot be drawn from the present data because it would rest on a single genotype call produced by a nonstandard assay and never resolved by phasing. TPMT*3A is defined by c.460G>A and c.719A>G in cis, whereas the same two variants in trans define TPMT*3B and TPMT*3C on opposite alleles and a poor-metabolizer phenotype, so the reported TPMT*1/*3A result remains clinically unresolved across an approximately tenfold difference in the recommended starting dose [11-13]. Any recommendation about the composition of a TPMT panel for Bangladeshi patients must therefore await DNA-based, phased confirmation in a larger series, and the present observation does no more than identify that question as one worth answering. Comparable departures from expected regional patterns have been reported from other understudied populations [26].

Current guidance converges on beginning 6-MP at approximately half the conventional dose in a confirmed intermediate metabolizer, with subsequent titration guided by tolerance and disease-specific protocols, and on an approximately 10-fold reduction with specialist input in a confirmed poor metabolizer [11,13]. We do not extend that guidance to the two patients identified here as though their phenotypes were established. For the patient reported as TPMT*1/*3C, a halved starting dose would be the guideline-concordant approach once the call is confirmed on genomic DNA. For the patient reported as TPMT*1/*3A, a halved starting dose could be substantially too high if the two variants lie in trans, so we regard DNA-based confirmation with allele-specific phasing as a prerequisite to any dose decision in that patient rather than an optional refinement; where phasing is unavailable, dosing should follow the more conservative of the two possible phenotypes under specialist supervision. Acting on genotype prospectively is far more informative than reacting to cytopenia, particularly during consolidation blocks in which several myelosuppressive agents are given together and the contribution of 6-MP cannot be isolated from blood counts alone.

Male patients predominated (77.5%), and B-lineage disease accounted for 60.0% of cases, which is consistent with the recognized male excess in ALL [7] and with the fact that most lymphoblastic malignancies arise from B-cell progenitors [1]. The post hoc comparisons of predicted metabolizer status by sex, immunophenotypic subtype, and categorized hematological parameters are reported in Tables 3, 4 for completeness and are not interpreted further. With two carriers, these analyses have no power; the absence of a detected difference carries no information in either direction, and germline TPMT variation would not in any case be expected to track disease phenotype at presentation.

Finally, TPMT is no longer the only determinant of thiopurine intolerance that matters in this region. Reduced-function NUDT15 variants are a major cause of mercaptopurine intolerance across Asian populations, and their effect is additive to that of TPMT [14,27,28]. NUDT15 variant carriers tolerate substantially lower 6-MP doses and face markedly higher leukopenia risk [15,29]; toxicity rates in Asian pediatric ALL cohorts are correspondingly high [30], and the NUDT15*3 allele has already been detected in Bangladeshi individuals [22]. NUDT15 was not assessed in the present study, formed no part of its objectives, and was not attempted, so the point that follows is derived entirely from the published literature cited above and not from our data. Based on that external evidence, TPMT genotyping alone captures only part of the pharmacogenetic risk in South Asian patients, and a combined TPMT and NUDT15 panel is likely the more rational testing strategy for Bangladeshi patients beginning 6-MP. Whether that expectation holds in this population remains to be tested.

This study has several strengths. It addresses a documented evidence gap: Bangladeshi TPMT data in ALL have been confined to one pediatric series, while pharmacogenetic testing is not yet routine, and patients were recruited consecutively at diagnosis in a unit where 6-MP-containing consolidation is delivered, so the population studied is the one in which pretreatment testing would apply. The analysis suits sparse data, using Clopper-Pearson exact intervals and Fisher exact and Fisher-Freeman-Halton tests rather than asymptotic alternatives, and the imprecision of the 5.0% estimate is stated plainly. However, this study had several limitations. First and most consequentially, genotyping used complementary DNA reverse transcribed from peripheral blood RNA rather than the genomic DNA specified by current recommendations, and no validation of it was performed, so the accuracy of every genotype call is unestablished. Because the laboratories returned diplotype calls without assay documentation, their accreditation, primers, variant panel, RNA integrity after transport, run controls, and calling criteria are unreported, and no sample was genotyped in duplicate or confirmed by a DNA-based method. Second, the protocol sample size calculation was invalid, with an allowable error of 0.05 against an anticipated proportion of 0.0276, defining no precision target, and has been withdrawn. The analyzed sample of 40 is a feasibility-limited consecutive series from one department, imprecise (exact 95% CI, 0.6-16.9), and unable to represent the national population. Third, without phasing, the TPMT*1/*3A patient cannot be distinguished from a TPMT*3B and TPMT*3C carrier in trans, a clinically decisive 10-fold rather than twofold dose difference. Fourth, testing was outsourced and paid for by participants, a plausible socioeconomic selection bias whose extent is unquantifiable because no enrollment log was kept. Fifth, the exploratory analyses had no power, and their p values are not evidence of independence. Sixth, genotyping was restricted to TPMT, so NUDT15 was not assessed, and age was not recorded, precluding age-stratified description in a disease with a bimodal age distribution. Finally, no prospective toxicity follow-up was undertaken, so the relationship between genotype and tolerance remains untested.

## Conclusions

Among 40 Bangladeshi patients with ALL, 38 had a reported TPMT*1/*1 diplotype and predicted normal metabolism, while two carried a reduced-function diplotype, TPMT*1/*3A and TPMT*1/*3C. No poor metabolizer was identified, although the TPMT*3A phase was unresolved and poor metabolism in that patient cannot be excluded. Because the calls came from an outsourced, unvalidated RNA-based assay, these frequencies are provisional and require DNA-based, phased confirmation before clinical use. The case for pretreatment TPMT testing rests on international guidance rather than these estimates, and combined NUDT15 testing follows from published Asian evidence, not from these data. Larger multicenter studies should as a minimum use accredited DNA-based genotyping with documented haplotype phasing and prospective toxicity monitoring, rather than replicating the present design.

## References

[1] Kantarjian H, Jabbour E (2025). Adult acute lymphoblastic leukemia: 2025 update on diagnosis, therapy, and monitoring. Am J Hematol.

[2] Malard F, Mohty M (2020). Acute lymphoblastic leukaemia. Lancet.

[3] Ni H, Shi Y, Wang M, Ji C (2025). Analysis of global, regional, and national burden and attributable risk factors of acute lymphoblastic leukemia and acute myeloid leukemia from 1990 to 2021. PLoS One.

[4] Huang P, Zhang J (2025). Global leukemia burden and trends: a comprehensive analysis of temporal and spatial variations from 1990-2021 using GBD (Global Burden of Disease) data. BMC Public Health.

[5] Hossain MS, Iqbal MS, Khan MA (2014). Diagnosed hematological malignancies in Bangladesh - a retrospective analysis of over 5000 cases from 10 specialized hospitals. BMC Cancer.

[6] Nahar K, Ferdousi SA, Hasan AR, Islam A, Islam A (2023). Incidence and outcome of childhood acute leukemia in a tertiary care hospital of Bangladesh armed forces. Dhaka Shishu Child Hosp J.

[7] Kakaje A, Alhalabi MM, Ghareeb A, Karam B, Mansour B, Zahra B, Hamdan O (2020). Rates and trends of childhood acute lymphoblastic leukaemia: an epidemiology study. Sci Rep.

[8] Toksvang LN, Lee SH, Yang JJ, Schmiegelow K (2022). Maintenance therapy for acute lymphoblastic leukemia: basic science and clinical translations. Leukemia.

[9] Krishnan S, Mahadevan A, Mungle T, Gogoi MP, Saha V (2024). Maintenance treatment in acute lymphoblastic leukemia: a clinical primer. Indian J Pediatr.

[10] Bayoumy AB, Ansari AR, Mulder CJ, Schmiegelow K, Florin T, De Boer NK (2024). Innovating thiopurine therapeutic drug monitoring: a systematic review and meta-analysis on DNA-thioguanine nucleotides (DNA-TG) as an inclusive biomarker in thiopurine therapy. Clin Pharmacokinet.

[11] Maillard M, Schwab M, Whirl-Carrillo M (2026). Clinical pharmacogenetics implementation consortium (CPIC) guideline for thiopurine dosing based on TPMT and NUDT15 genotypes: 2025 update. Clin Pharmacol Ther.

[12] Pratt VM, Cavallari LH, Fulmer ML (2022). TPMT and NUDT15 genotyping recommendations: a joint consensus recommendation of the Association for Molecular Pathology, Clinical Pharmacogenetics Implementation Consortium, College of American Pathologists, Dutch Pharmacogenetics Working Group of the Royal Dutch Pharmacists Association, European Society for Pharmacogenomics and Personalized Therapy, and Pharmacogenomics Knowledgebase. J Mol Diagn.

[13] Coenen MJ, Nijenhuis M, Soree B (2026). Dutch Pharmacogenetics Working Group (DPWG) guideline for the gene-drug interaction between TPMT/NUDT15 and thiopurines. Eur J Hum Genet.

[14] Maillard M, Nishii R, Yang W (2024). Additive effects of TPMT and NUDT15 on thiopurine toxicity in children with acute lymphoblastic leukemia across multiethnic populations. J Natl Cancer Inst.

[15] Du S, Huang X, He X (2024). Association of NUDT15 gene polymorphism with adverse reaction, treatment efficacy, and dose of 6-mercaptopurine in patients with acute lymphoblastic leukemia: a systematic review and meta-analysis. Haematologica.

[16] Zhou Y, Lauschke LM, Lauschke VM (2026). Comprehensive overview of the global pharmacogenetic and functional variability in NUDT15. Hum Genomics.

[17] Ranasinghe P, Liyanage C, Sirisena N, Liyanage S, Priyadarshani CD, Hendalage DP, Dissanayake VH (2024). Frequency of pharmacogenomic variants affecting safety and efficacy of immunomodulators and biologics in a South Asian population from Sri Lanka. Hum Genomics.

[18] Singh A, Pandita RM, Goyal MK, Dhillon BK, Midha V, Sood A (2026). Decoding genetic divergence: TPMT and NUDT15 polymorphisms in north India mirror Caucasian ancestry. Intest Res.

[19] Zaman S, Fukushima H, Suzuki R, Yoshimatsu S, Hawlader MDH, Fukushima T (2019). TPMT and ITPA gene polymorphism and their adverse events during chemotherapy of acute lymphoblastic leukemia among Bangladeshi children. Iran J Blood Cancer.

[20] Rashid MMU, Ahmed I, Islam MdA (2020). Influence of TPMT polymorphisms on azathioprine-induced myelosuppression in Bangladeshi patients with systemic lupus erythematosus. Drugs Ther Perspect.

[21] Karim MM, Hassan MM, Rahaman MFU, Azad MAK, Rahman A, Islam MN (2026). The frequency of thiopurine methyl-transferase polymorphism in systemic lupus erythematosus patients and its association with azathioprine induced adverse effects. Bangladesh J Med.

[22] Sarker DB, Akash MdMH, Sajib AA, Akhteruzzaman S (2022). Identification of NUDT15*3 variant in the Bangladeshi population using allele-specific PCR. ReGEN Open.

[23] Mostaid MS, Aziz MA, Maisha JA, Islam MS, Maruf AA (2023). A review of pharmacogenetic studies in the Bangladeshi population. Drug Metab Pers Ther.

[24] Murugesan R, Vahab SA, Patra S (2010). Thiopurine S-methyltransferase alleles, TPMT*2, *3B and *3C, and genotype frequencies in an Indian population. Exp Ther Med.

[25] Stachelski J, Pham D, Hughes P, McLachlan O, Dervieux T (2023). B-225 assessment of the genotype frequency of thiopurine methyltransferase (TPMT) deficiency in a large cohort of patients with immune mediated inflammatory disease and cancer. Clin Chem.

[26] Chikondowa P, Munkombwe D, Chikwambi Z, Kuona P, Masimirembwa C (2023). Pharmacogenetics of 6-mercaptopurine in a black Zimbabwean cohort treated for acute lymphoblastic leukaemia. Pharmacogenomics.

[27] Khaeso K, Udayachalerm S, Komvilaisak P (2021). Meta-analysis of NUDT15 genetic polymorphism on thiopurine-induced myelosuppression in Asian populations. Front Pharmacol.

[28] Lubis IS, Anggadiredja K, Artarini AA, Sari NM, Suryawan N, Zazuli Z (2025). NUDT15 pharmacogenetics in acute lymphoblastic leukemia: synthesizing progress for personalized thiopurine therapy. Med Sci (Basel).

[29] Yu N, Sriranganathan D, Walker GJ (2023). Prevalence of NUDT15 genetic variants and incidence of thiopurine-induced leukopenia in inflammatory bowel disease: a systematic review and meta-analysis. J Crohns Colitis.

[30] Rosdiana DS, Saputri DS, Louisa M, Setiabudy R (2024). NUDT15 polymorphism and its association with mercaptopurine hematotoxicity in acute lymphoblastic leukemia in Indonesian children. In Vivo.

